# The Role of Human Milk Immunomodulators in Protecting Against Viral Bronchiolitis and Development of Chronic Wheezing Illness

**DOI:** 10.3390/children2030289

**Published:** 2015-07-07

**Authors:** Dani-Louise Dixon

**Affiliations:** 1 Intensive and Critical Care Unit, Flinders Medical Centre, Adelaide 5042, Australia; E-Mail: dani.dixon@flinders.edu.au; Tel.: +61-8-8204-5494 (ext. 65494); Fax: +61-8-8204-5751; 2 Department of Critical Care Medicine, Flinders University, Adelaide 5001, Australia

**Keywords:** bronchiolitis, RSV, viruses, breastfeeding, leukocytes, cytokines

## Abstract

Infants who are breastfed are at an immunological advantage when compared with formula fed infants, evidenced by decreased incidence of infections and diminished propensity for long term conditions, including chronic wheeze and/or asthma. Exclusive breastfeeding reduces the duration of hospital admission, risk of respiratory failure and requirement for supplemental oxygen in infants hospitalised with bronchiolitis suggesting a potentially protective mechanism. This review examines the evidence and potential pathways for protection by immunomodulatory factors in human milk against the most common viral cause of bronchiolitis, respiratory syncytial virus (RSV), and subsequent recurrent wheeze in infants. Further investigations into the interplay between respiratory virus infections such as RSV and how they affect, and are affected by, human milk immunomodulators is necessary if we are to gain a true understanding of how breastfeeding protects many infants but not all against infections, and how this relates to long-term protection against conditions such as chronic wheezing illness or asthma.

## 1. Introduction

The development of the infant immune system is influenced by primary exposure to antigens that will promote either antibody dominant humoral immune responses (T helper lymphocyte (Th)2 mediated) or cytokine-cell mediated responses (Th1 mediated) [1,2,3,4]. The switch from Th2 to a Th1/Th2 balance may be affected by many factors including primary exposure to either Th1 or Th2 promoting antigens or mode of feeding [1,2,3,4,5,6]. Th1-type responses are likely to be stimulated by non-pathogenic bacteria and some viral infections. However, in current western environments very young infants are exposed to fewer immunogenic organisms than previous generations. Many significant antigens now encountered in early infancy are allergens, such as house dust mite and animal dander, and viruses such as respiratory syncytial virus (RSV), all of which have the potential to promote a Th2 polarised response.

Infants who are breastfed are at an immunological advantage when compared with formula fed infants, as evidenced by both decreased incidence of infections during infancy but also diminished propensity for a number of long term conditions, including chronic wheeze and/or asthma [7,8,9,10]. These advantages indicate a biological link between a mother and her infant via the factors supplied in the milk. While the transfer of a number of factors in human milk including immunoglobulins, lactoferrin and oligosaccharides provide passive protection for the recipient infant, it may be speculated that alterations to the propensity for chronic wheezing illness are more likely due to factors capable of modulating the development of the infant immune system [7,11].

Potential immunomodulators which continue to be identified in human milk include most established mediators, such as cytokines and maternally derived leukocytes, but may also include epigenetic factors. These immunomodulators may affect development through both direct and indirect influences on the naive cells of the immature infant immune system during the early, formative stages of immune development (Table 1).

**Table 1:** Immunomodulators measurable in expressed breast milk (EBM) and potential actions within the recipient infant.

	Examples in EBM	General Effects	Possible effects during RSV infection
Pro and anti-inflammatory	TNF-α, IL-1, PGE_2_ IL-10, TGF-β, TXA_2_. miRNA’s	Vasodilation, synthesis of acute phase proteins, stimulation of B & T cells and monocytes Vasoconstriction, downregulation of inflammatory mediators, oral tolerance	SMC stimulation, bronchoconstriction, production of chemokines, cellular influx Control of inflammatory cascade, apoptosis of inflammatory cells
Leukocyte differentiation	GM-CSF, M-CSF IFN-γ, TGF-β, IFN-β IL-2, IL-4, IL-6, miRNA’s	Monocyte differentiation to M1 or M2 Neutrophil differentiation to N1 or N2 Lymphocyte differentiation to Th1, Th2 or Th17, Tregs and B cell differentiation	Viral clearance, tissue repair or damage Resolution or repair, tissue damage, fibrosis, stiffening & hypersensitivity Regulate disease pathway & memory
Tissue fibrosis and repair	TGF-β, KGF, HGF	Growth factor production, fibrosis Epithelial & endothelial tissue repair	Airway thickening, tissue stiffening Healthy epithelial tissue repair
Chemotactic	IL-8, TNF-α, TXA_2_ MCP-1, LTB_4_ RANTES	Neutrophil attraction and tissue migration Monocyte/macrophage attraction & migration T lymphocyte migration	Leukocyte activation and infiltration, secondary mediator release, phagocytosis and viral clearance, promotion of inflammatory cascade or tissue repair
Atopic	IL-2, IFN-γ, IL-12 IL-4, IL-5, IL-13	T cell stimulation to Th1 T cell stimulation to Th2, eosinophil attraction, stimulation of IgE production	Th cell acquired immunity, & memory Airway hypersensitivity
Antibody production	TNF-α, IL-6, TGF-β IL-4	Isotype switching, stimulus of IgA production Isotype switching, stimulus of IgE production	RSV specific IgA production Generalised hypersensitivity
Leukocytes	Polymorphonuclear Monocytes/macrophages Lymphocytes	Degranulation, epithelial damage, phagocytosis Phagocytosis, inflammatory resolution & repair Link innate and acquired responses	Prolonged epithelial damage & fibrosis, atopic activation Viral clearance, tissue repair or prolonged inflammation and fibrosis Immunological memory, atopic hypersensitivity, cytotoxicity & tolerance

## 2. Infant Immune Activation

Th2-type cytokines are predominant in favourable intrauterine environments of human infants [8]. Studies have demonstrated that a mother’s mononuclear cell population expressing decreased levels of interferon (IFN) -γ and interleukin (IL) -2 (Th1 cytokines), combined with an increase in IL-4 and IL-10 (Th2 cytokines) is indicative of successful pregnancy [8,9]. It has been postulated that healthy development of the infant immune system therefore requires a “switch” from the Th2 biased intrauterine environment to a balanced Th1/Th2 immune response *ex utero*.

Infants born to atopic mothers are at higher risk of developing hypersensitivity than those born to an atopic father [10]. This may be due to exacerbation of the Th2 dominated intrauterine environment in atopic mothers, as demonstrated by higher levels of amniotic fluid IL-10 and immunoglobulin (Ig) E in these mothers [12]. As early as week 8 of gestation, the foetal thymus contains functional T lymphocytes and by week 11, there are B lymphocytes present in foetal tissues that are capable of producing IgE [13,14,15]. This indicates a substantial maturation of the infant immune system during exposure to this highly Th2 predominant environment that may be further skewed by genetic or epigenetic factors predisposing to atopy. The effects of this may be demonstrated in part by the inhibition of IFN-γ production by cord and peripheral blood mononuclear cells (PBMC) and the expression of IL-13 (Th2 cytokine) mRNA by ovalbumin stimulated PBMCs of infants at birth, who later present with food-associated atopic eczema [16,17]. In this context epigenetics has been hypothesized as of high importance because of the concept that external factors may possess the ability to alter gene expression without modifying the DNA sequence. Genetic materials identified in human milk including microRNAs, stem cells and substances such as lactoferrin as well as nutritional components have all been either demonstrated or been postulated to affect epigenetic regulation and gene transcription [18,19]. As such, the greater maternal influence on propensity toward atopy or wheeze may be due to postnatal differences in environment, including the transfer of immunity via maternal milk. 

Children exposed to early infections, other than those of the lower respiratory tract, through contact with older siblings or attendance at day care or nursery facilities, prior to 6 months of age, are less likely to develop asthma than their only child, home care peers [20,21,22,23]. Alternately, daily intake of non-pathogenic bacteria such as *Lactobacillus rhamnosus* (Lactobacillus GG) to mothers in the final weeks of pregnancy and continuing through 6 months of breastfeeding can halve the incidence of atopy during the first 2 years of an infant’s life [24], while direct administration of antigens associated with diseases such as measles, whooping cough, influenza and tuberculosis through either vaccination or natural infection during infancy, is associated with decrease in the incidence of atopic symptoms in older children (13–14 years) [25,26,27,28]. These antigens are all known to primarily induce Th1-type, IFN-γ-rich responses by the immune system, which in turn suppress IL-4 expression resulting in IL-12-mediated Th1 cell development and inhibition of Th2 cell development [29]. However, in the absence of these Th1 promoting antigens, the primary exposure of infants is most likely to be to allergens, as well as viruses predominant in childhood illness such as RSV.

## 3. Respiratory Syncytial Virus

A link between hospitalisation with RSV bronchiolitis and the subsequent development of chronic wheezing illness has been well established [3,30,31,32]. RSV is the most common childhood respiratory pathogen, infecting approximately 90% of all infants in the first 12–18 months of life. Of those infected, approximately 3% require hospitalisation due to the onset of severe bronchiolitis and of these infants, between 23% and 75% go on to develop childhood asthma [5,30,33]. This makes RSV infection one of the most important causal factors for the development of recurrent wheezing illness in the developed world, where allergy incidence is rapidly increasing [34,35,36,37,38].

The mechanism by which RSV predisposes to chronic wheeze or asthma is unclear. One hypothesis centres around the extensive damage caused to the respiratory epithelium by the virus via upregulation of chemokines such as IL-8 and regulated on activation, normal T cell expressed and secreted (RANTES), which results in an influx of polymorphonuclear granulocytes, particularly neutrophils and eosinophils, into the airways [39,40,41,42,43]. Degranulation of these granulocytes leads to increased sensory nerve stimulation or triggering of reflex bronchoconstriction resulting in airway hyperresponsiveness and persistent wheeze [44,45], and may also increase airway epithelial permeability to allergens [46]. Other hypotheses focus on the potential contribution of RSV to heightened allergen sensitisation due to production of Th2 and other cytokines such as RANTES, macrophage inflammatory protein (MIP)-1α and IFN-γ by the chemokine-recruited leukocytes, leading to the production of RSV specific IgE [42,43,44]. A murine model of RSV infection has shown that this initial cytokine response can lead to increased airway responsiveness with eosinophilia and neutrophilia associated with a resultant increase in Th2 cytokine production upon exposure to allergen [47]. A prospective cohort study of infants previously hospitalised with RSV bronchiolitis against age and sex matched controls also demonstrated a significant increase in subsequent total serum IgE levels, as well as a significantly greater prevalence of asthma [33]. However, a definitive causal relationship between RSV infection and the development of atopic disease has yet to be elucidated.

### 3.1. RSV Th1 and Th2 Type Effects

Activation of the immune system by infections such as RSV and autoimmune diseases such as atopy, significantly alter the cytokine profiles produced by immunoactive cells. These changes affect cells not only at sites of activation, such as the respiratory mucosal surfaces, but also systemically in peripheral blood. The peripheral blood mononuclear cells of infants hospitalised with RSV-mediated respiratory tract infections demonstrate a significantly elevated IL-4 to IFN-γ ratio upon mitogen stimulation *in vitro* [48].

RSV infection also alters ratios of lymphocyte subsets through an increase in B lymphocyte numbers and changes in CD8^+^ T lymphocytes (cytotoxic/suppressor) and CD8^+^/CD25^+^ (IL2 receptor) activated T lymphocyte populations [48,49,50,51]. Cellular depletion experiments using a murine model of RSV infection identified CD4^+^ (helper/inducer) T lymphocytes as being the primary effectors responsible for viral clearance rather than the CD8^+^ cytotoxic subset [52]. Murine studies have shown that IL-12 is able to abrogate RSV disease through decreasing IL-4 and IL-5 and increasing IFN-γ production, while treatment with anti-CD8 or anti-IFN-γ antibodies increase disease manifestation in RSV infection by enhanced Th2- driven eosinophilia [53,54].

RSV infection upregulates both IL-10 mRNA and protein expression by lung alveolar macrophages, as well as increasing the ratio of IL-4/IFN-γ production by PBMC [48,55,56]. These changes have the potential to promote the infiltration and activation of lymphocytes to a Th2 type phenotype, the production of Th2 type cytokines, antibody isotype switching to IgE and significant lung eosinophilia [53]. Restoration of the balance between Th2/Th1 by depletion of IL-4 or administration of recombinant IL-12 increases cytotoxic CD8^+^ T lymphocyte or CD4^+^ T lymphocyte activity, respectively, both resulting in increased viral clearance and reduced severity of illness in RSV-infected mice [52]. Intramuscular recombinant IL-12 administration also resulted in a significant decrease in viral titre in the lung tissue of RSV-infected mice using this model. However, the administration of this Th1 cytokine in the cellular depletion experiments identified CD4^+^ T lymphocytes as the major effector for viral clearance. This indicates a differing immunological mechanism working under the influence of Th1 *versus* Th2 cytokines in RSV infection.

It is therefore feasible that maternal RSV infection will exert similar effects on the cells and cytokines found in human milk, manifesting in changes to the Th1/Th2 balance and ultimately in differing responses in the infant.

### 3.2. RSV and Breastmilk

Breastfeeding confers protection against both incidence and severity of RSV disease, particularly in those born prematurely, as well as the subsequent development of recurrent wheezing illness [57,58,59,60,61,62,63,64,65]. The intake of human milk with all of its immunomodulatory constituents may contribute to protection against both the occurrence and consequences of RSV disease either by non-specifically enhancing or “maturing” the immune system of the recipient infant, or by exerting some means more specific to RSV itself. However it does seem clear that degree, or exclusivity, of breastfeeding may affect RSV disease outcome, if not absolute infection rate. Exclusive breastfeeding reduces the duration of hospital admission, risk of respiratory failure and requirement for supplemental oxygen in hospitalised bronchioltic infants [66,67]. These decreases in disease severity correlate with decreased airway chemokine concentration, expression of activation markers and inflammatory cellular infiltrate in exclusively but not partially breastfed infants [67,68]. These exclusivity studies raise the question of whether temporal dose of human milk immunomodulators or conversely exposure to formula milk constituents are key.

Breastfed infants with RSV infection have markedly higher serum IFN-α levels than RSV infected non-breastfed infants during the course of the illness and continuing throughout the first 2–4 weeks post-infection [69]. This may indicate a heightened immune response in these infants. However, no difference in production of IFN-α has been observed between infants who had been weaned from human milk when compared with those bottle fed from birth, suggesting only an immediate benefit which disappears at cessation of breastfeeding [69]. In contrast, length of breastfeeding regardless of time since cessation has been associated with decreased length of bronchiolitic illness in parallel to a weak negative association with airway concentration of the neutrophil chemokine IL-8 [67].

The lymphocyte population of human milk consisting largely of T cells is made up of significantly more activated (HLA-DR^+^ and CD25^+^), and memory (CD45RO^+^) T cells than peripheral blood [70,71,72,73]. These lymphocytes also produce a number of cytokines *ex vivo* [74,75]. The cell surface molecules CD30 and CD40 ligand (CD40-L), both members of the TNF receptor family, are upregulated on colostral T cells compared to peripheral blood T cells [76,77]. The increased expression of cell surface molecules such as CD40-L on colostral cells, in the absence of CD40-L expression on infant cells, may be direct evidence of a compensatory function of these human milk lymphocytes [78]. Studies examining human milk cell responses to RSV have demonstrated proliferation comparable to that of PBMC in response to exposure to virus *in vitro*, indicating a comparable response by human milk-derived leukocytes to that from circulatory leukocytes [79].

Human milk cells primed by exposure of the mother to RSV in the general population may carry a bias toward Th2 cytokine expression that may be carried into the system of the breastfed infant [80]. High concentrations of potentially immunomodulatory cytokines and live, active, cells in human milk, have been demonstrated, some of which are modified through maternal RSV exposure [80,81], or alternately can modify the epithelial cytokine response [82]. Therefore, infant exposure to RSV likely results in the stimulation of cells ingested in human milk which may then be carried to sites of immune activation, either directly such as the Peyers patches in the gastrointestinal mucosa or via the circulation to the lymph nodes, bone marrow, spleen and thymus. A predisposition toward Th2 cytokine production by activated human milk cells combined with an environment of Th2 predominant secreted cytokines has the potential to stimulate infant mononuclear cells to Th2-type cell differentiation. Bias toward Th2-type cell development would facilitate induction of B lymphocyte immunoglobulin isotype switching to IgE production upon encounters with allergens. This may therefore indicate that predominance of Th1 promoting factors in human milk may result in the prevention of the development of hypersensitivity reactions or inappropriate immune responses in recipient infants.

Alternately, factors which may regulate the production of chemotactic cytokines and subsequent neutrophil chemotaxis, as well as neutrophil activity are also prevalent in human milk [83]. Lactoferrin, present in human milk in 1000-fold greater concentration than that of plasma [81], decreases epithelial production of chemokines [82,84] as well as diminishing the response to these chemokines by recruited neutrophils [85] while milk-derived oligosaccharides and procathepsin D alter neutrophil adhesion molecule CD11b and L-selectin expression [86,87,88], thereby potentially decreasing both neutrophil infiltration and activation in the airways.

Dendritic cells (DCs) prevalent in both the gastrointestinal and respiratory mucosa provide an intrinsic link between innate and adaptive immunity which is substantively regulated by the microenvironment at the time of antigen recognition. Resultant differences cytokine and in cell surface molecule expression, including toll-like receptors (TLRs), determine DC effects on the development of T-cell sub-populations (Th1/Th2/Th17 and regulatory T cells) and thereby antigen-specific activity by T cells. While mature DC have not been identified in human milk, the potential for breast milk macrophages to commence this transition has been demonstrated *ex vivo* [89]. These breast milk macrophages were found to represent an intermediate stages of cell differentiation between that of a peripheral blood macrophage and a fully differentiated DC. However, factors with the potential to regulate the activation of DC have been identified in human milk. Human milk glycans, such as those expressed in oligosaccharides or on glycoproteins, bind to the C-type lectins receptors on DC and specific tissue macrophages thereby preventing pathogen interaction. Human milk MUC1 has been identified as a major milk glycoprotein that binds to DC-specific intercellular adhesion molecule-3-grabbing non-integrin (DC-SIGN), a constitutive receptor within the gastrointestinal tract of infants, suggesting the importance of human milk glycoproteins for blocking pathogen interaction to DC in recipient infants [90]. As RSV infection mobilizes myeloid and plasmacytoid DCs to the airways, the transfer of these factors to the recipient breastfed infant has the potential to affect outcomes both to RSV disease acutely but also to the subsequent development of hypersensitivity [91].

An association between the microbiota of human milk and of the infant gut has been established [92,93]. More recently evidence has emerged for a link between maternal milk microbiome and colonization of the infant nasopharynx [94]. Breastfed infants were found to have increased prevalence of *Corynebacterium* and *Dolosigranulum* a newly recognized member of the family of lactic acid bacterium, generally considered to be contributors to a healthy microbiota. Conversely formula fed infants had decreased *Staphylococcus*, and *Prevotella* and *Veillonella,* anaerobic bacteria, recently associated with an increased risk of otitis media [95]. While this study was not adequately powered to assess clinical outcomes an inverse association between the prevalence of *Dolosigranulum* in the nasopharynx and parent reported symptoms of wheezing and respiratory infections was observed. The reported effect was absent by 6 months of age suggesting a limited window of direct benefit. However, this study provides an interesting beginning to this novel area of investigation and does not preclude the possibility for early immunomodulation by favorable microbiota extending beyond this window of difference.

Finally, miRNA expression has been established in human milk, including identification of miRNA’s unique to milk and large quantities of immune-related miRNAs particularly in the first six months postpartum [19,96,97]. As the field of epigenetics is relatively new, research in relation to bronchiolitis and RSV infection has so far been largely *in vitro* however a single clinical study describing differentiation of miRNA response not just to RSV infection but also relating to disease severity marks this as a promising novel avenue for the future [98].

### 3.3. Maternal-Infant Communication in RSV

A link between infant and mother through milk feeding is well accepted. However, evidence supporting the potential for that link to be bi-directional has only recently emerged. Maternal exposure to RSV during their infant’s disease course significantly increases the number of cells passed from the mother to her infant in milk [80]. In addition, these cells differ from the cells of non-exposed mothers’ in response to re-exposure to RSV through production of an elevated Th2:Th1 (IL-10:IFN-γ) cytokine profile. This effect is unique to stimulation with live RSV, not when stimulated with a non-specific mitogen (Concanavalin A) and is reflected in peripheral blood cells from RSV infected infants [48]. Skewed cytokine responses to RSV by the milk cells from mothers of bronchiolitic infants may indicate that these cells have homed to the mammary gland following activation by the virus within the mucosa-associated lymphoid tissue in the respiratory tract of the mother. This response implies that not only are the cells of breastfeeding mothers immunologically activated by exposure to their infants illness, but that this activation, in the case of RSV bronchiolitis, may result in homing of large numbers of virus activated cells to the mammary gland from where they will then be passed on to the infant, completing the cycle.

This research provides evidence for a direct link between the cells found in human milk and illness in the recipient infant, regardless of the presence of clinical symptoms in the breastfeeding mother. The marked increase in cells, which are skewed to a Th2 cytokine response to RSV, may affect the outcomes of RSV disease and the development of asthma in the recipient infant. Determining the degree and specificity of activation of human milk cells that traverse the mammary gland during RSV infection will help determine the potential for these cells in activating and modulating the developing immune system of the recipient infant.

## 4. Conclusions

Further investigations into the mechanisms by which respiratory viruses such as RSV affect human milk immunomodulators is necessary if we are to gain a true understanding of how breastfeeding protects many infants against infections, as well as associated long-term immune-mediated conditions such as chronic wheezing illness or asthma. This future research must also examine why, for some infants this protection is incomplete. In this light, the composition of human milk must be profiled further. This is a continuing process with new cytokines being identified in samples of human milk almost as soon as they are identified in blood. Similarly, the cells of human milk are currently being identified and characterised by many researchers and this process must continue, including broadening to encompass the latest understanding of leukocyte cell subsets such as the T cell Treg and Th17 and monocyte/macrophage M1 and M2 if we are to understand the potential influence of these potent immunomodulators.

Identifying the potential of these human milk cells, as well as the complete cytokine profile of milks in response to immuno-active conditions, will also provide the first steps toward furthering research into modification of these factors. While cellular activation, as well as the production of many cytokines and prostaglandins, can be regulated by pharmaceuticals, in the context of infant feeding a preferable option would always be less synthetic means such as nutriceuticals including the pro-biotics [24,99,100]. The administration of “friendly” bacteria, either with human milk as supplements, or through the milk by supplementing the mother [101], shows tremendous potential benefits in addressing the limitations in infant immune development outlined by the “hygiene hypothesis” [102].

Overall, we have merely scratched the surface in the process of gaining an understanding of what is in milk, how it gets there and what it does in the context of respiratory viral illnesses so often shared by mother and child. Extensive further research is required if we are ever to truly appreciate the benefits available to all infants via this paradoxical fluid.
